# Expression, purification and crystallization of the photosensory module of phytochrome B (phyB) from *Sorghum bicolor*

**DOI:** 10.1107/S2053230X24000827

**Published:** 2024-02-20

**Authors:** Sintayehu Manaye Shenkutie, Soshichiro Nagano, Jon Hughes

**Affiliations:** a Department of Plant Physiology, Justus Liebig University Giessen, Senckenbergstrasse 3, 35390 Giessen, Germany; b Department of Chemistry, Hawassa University, PO Box 05, Hawassa, Ethiopia; UNSW Sydney, Australia

**Keywords:** *Sorghum bicolor*, phytochrome B, maturity, crystallization, diffraction quality

## Abstract

Sorghum, a short-day tropical plant, has been adapted for temperate grain production, in particular through the selection of variants at the *MATURITY* loci (*Ma1*–*Ma6*) that reduce photoperiod sensitivity. *Ma3* encodes phytochrome B (phyB), a red/far-red photochromic biliprotein photoreceptor. The multi-domain gene product, comprising 1178 amino acids, autocatalytically binds the phytochromobilin chromophore to form the photoactive holophytochrome (Sb.phyB). This study describes the development of an efficient heterologous overproduction system which allows the production of large quantities of various holoprotein constructs, along with purification and crystallization procedures. Crystals of the Pr (red-light-absorbing) forms of NPGP, PGP and PG (residues 1–655, 114–655 and 114–458, respectively), each C-terminally tagged with His_6_, were successfully produced. While NPGP crystals did not diffract, those of PGP and PG diffracted to 6 and 2.1 Å resolution, respectively. Moving the tag to the N-terminus and replacing phytochromobilin with phycocyanobilin as the ligand produced PG crystals that diffracted to 1.8 Å resolution. These results demonstrate that the diffraction quality of challenging protein crystals can be improved by removing flexible regions, shifting fusion tags and altering small-molecule ligands.

## Abstract 2

A heterologous holophytochrome overproduction system has been developed to produce large quantities of three holoprotein constructs of phytochrome B from *S. bicolor* for crystallization. The results showed that the diffraction quality of the crystals could be improved by removing flexible regions, shifting the fusion tag and changing the type of ligand.

## 1. Introduction

*Sorghum bicolor* is a drought-tolerant, multipurpose crop that can be used as a food, feed and fuel. It is a tropical, short-day-flowering species with substantial photoperiod sensitivity. Given its fundamental impact on sorghum yield, photoperiodic flowering induction has been a key focus of sorghum breeding programs that have led to the development of early maturing, photoperiod-insensitive and dwarfed temperate-adapted sorghum cultivars with improved yield. These cultivars harbour mutations in six *MATURITY* loci (*Ma1*–*Ma6*; Childs *et al.*, 1992 ▸), with *Ma3* encoding the phytochrome B (phyB) apoprotein (Childs *et al.*, 1997 ▸).

Phytochromes are red (R)/far-red (FR) photochromic biliprotein photoreceptors that are found in plants, bacteria and fungi. They play pivotal roles in controlling a diverse array of biological processes, encompassing developmental transitions such as germination, seedling photomorphogenesis, floral induction and stress responses. Phytochrome apoproteins are characterized by their bilin lyase activity to form holophytochromes, whereby the bilin chromophore is covalently attached to a conserved cysteine residue (Rockwell & Lagarias, 2010 ▸). Bilin biosynthesis involves oxidative cleavage of the heme cofactor into biliverdin (BV), a reaction catalysed by heme oxygenase (HO). BV is subsequently converted into phycocyanobilin (PCB) or phytochromobilin (PΦB) through the action of ferredoxin-dependent bilin reductases (FDBRs) in cyanobacteria and plants (Kohchi *et al.*, 2001 ▸), respectively. Plant holophytochromes undergo photoconversion between the physiologically inactive R-absorbing Pr state and the FR-absorbing signalling state, Pfr (Butler *et al.*, 1959 ▸). This R/FR photoreversibility allows physiological responses induced by R to be nullified by subsequent FR treatment. Phytochromes, which function as light-regulated signalling proteins, participate in complex signal transduction pathways that orchestrate intricate developmental and physiological responses.

**Figure 1:**
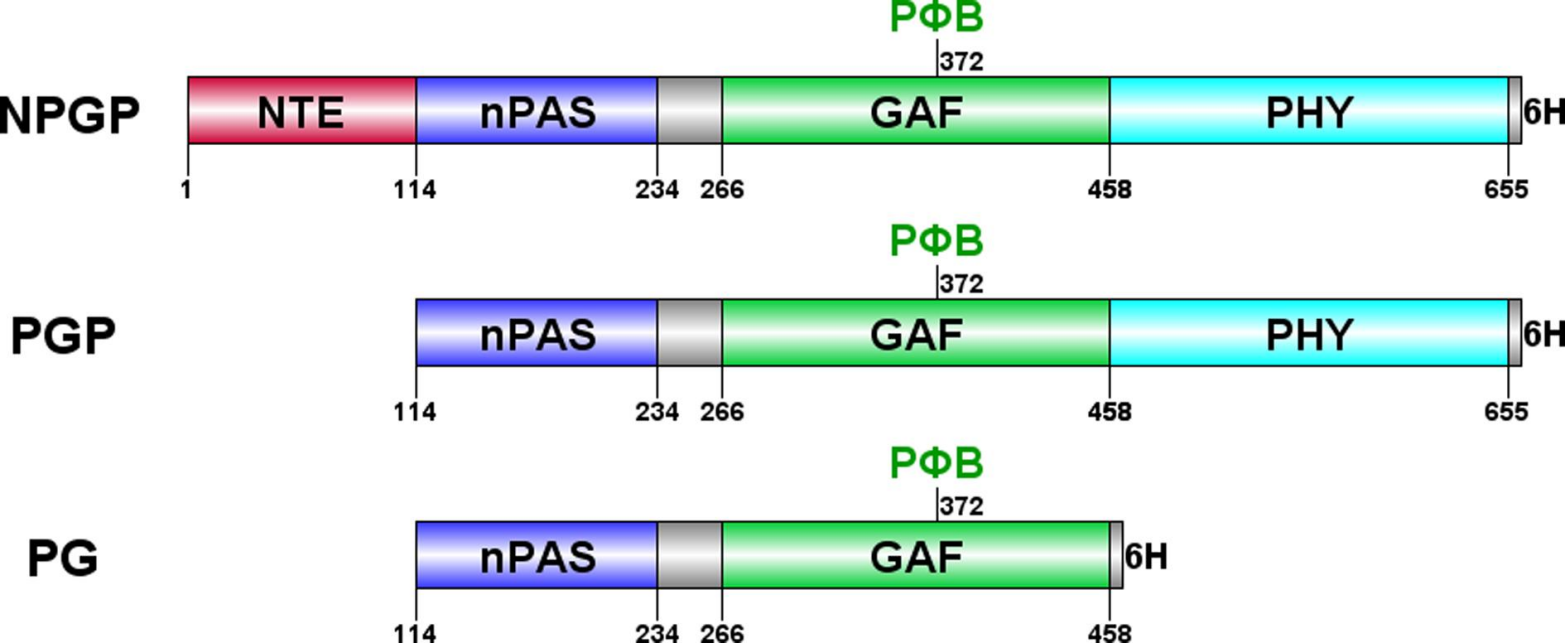
Domain structure of the photosensory module (PSM) of *Sorghum* phyB. NPGP comprises the NTE and the nPAS, GAF and PHY domains. PGP lacks the NTE, whereas PG lacks both the NTE and the PHY domain. The phytochromobilin (PΦB) chromophore is covalently bound to Cys372. In all of these constructs, the C-terminal module has been removed and a His_6_ tag has been appended.

The N-terminal photosensory module (PSM) of *S. bicolor* phyB, as illustrated in Fig. 1 ▸, is composed of an N-terminal extension (NTE), a Per/Arnt/Sim (nPAS) domain, a cGMP-specific phosphodiesterase/adenylyl cyclase/FhlA (GAF) domain and a phytochrome-specific (PHY) domain. Notably, the NTE contains a low-complexity region. Within the PSM, the GAF domain forms the conserved bilin-binding pocket, with the chromophore covalently attached at Cys372 to form the holoprotein. The PSM of phytochromes is distinguished by a figure-of-eight knot that ties the GAF and nPAS domains together (Wagner *et al.*, 2005 ▸), a helical spine linking the GAF and PHY domains (Essen *et al.*, 2008 ▸) and a tongue-like hairpin extending from the PHY domain to contact the GAF domain (Essen *et al.*, 2008 ▸). The tongue undergoes radical conformational changes upon Pr/Pfr photoconversion (Takala *et al.*, 2014 ▸).

Given the pivotal role of phytochromes in plant growth and development, the prospect of structure-guided engineering of phyB holds immense potential for enhancing agronomic traits in crops. Furthermore, it is noteworthy that the PSM is both necessary and sufficient for light perception, photoconversion and signalling in plants (Matsushita *et al.*, 2003 ▸), with the downstream domains being required for dimerization and intracellular translocation. Consequently, our objective was to elucidate the structural details of the PSM of *Sorghum* phyB, initially in its Pr state. Here, we document our success in the overproduction, purification and crystallization of three distinct PSM constructs in the Pr state. Two of these crystals have produced high-resolution structures, which are now available in the Protein Data Bank (PDB) as entries 6tc5 and 6tby, and have been published (Nagano *et al.*, 2020 ▸).

## 2. Materials and methods

### 2.1. Construction of expression plasmids

The native *S. bicolor* phyB cDNA (GenBank accession No. AF182394) was codon-optimized for expression in *Escherichia coli* using *Gene Designer* 2.0. The folding free energy of the mRNA head region was optimized to avoid secondary structure (Gruber *et al.*, 2008 ▸). A synthetic gene encoding the first 655 residues corresponding to the PSM together with a ribosome-binding site (RBS) was synthesized and inserted into the EcoRV site of the pUC57 plasmid by Eurofins Genomics (Ebersberg, Germany).

Domain boundaries were established based on the crystal structure of a cyanobacterial phytochrome (Essen *et al.*, 2008 ▸) and secondary-structure predictions. Deletion constructs were produced by PCR using Phusion High-Fidelity DNA Polymerase (New England Biolabs). The DNA sequences encoding the NPGP-H_6_ (residues 1–655 with a His tag) and PGP-H_6_ (residues 114–655 with a His tag) constructs, as provided in Supplementary Table S1, were generated using the primers listed in Supplementary Table S2 (GATC Biotech, Konstanz, Germany). The upstream and downstream primers, which encode an in-frame ATG codon and a His-tag-coding sequence with a termination codon, respectively, were used to incorporate proximal EcoRI and distal HindIII restriction sites into the PCR products. The resulting amplicons were digested with EcoRI and HindIII and ligated into the pPROLar.A plasmid. The pPROLar.A expression plasmid is a component of the PRO Bacterial Expression System developed by Lutz & Bujard (1997 ▸). The procedures for cloning the genes encoding heme oxygenase 1 (*HO1*) and *pcyA* from *Synechocystis* 6803 into plasmid p171, as well as the *Synechocystis* *HO1* gene and the *Arabidopsis thaliana* PΦB synthase (*HY2*) gene into plasmid p183, have been described previously (Landgraf *et al.*, 2001 ▸).

### 2.2. Expression using the pPROLar.A vector

To produce holophytochromes by *in vivo* assembly with PΦB, the pPROLar Sb.phyB constructs (pPROLar-NPGP-H_6_ and pPROLar-PGP-H_6_) were cotransformed with plasmid p183 for PΦB production into competent *E. coli* BL21_PRO_ cells (Clontech). The transformed cells were selected on lysogeny broth (LB) plates containing ampicillin (100 µg ml^−1^) and kanamycin (50 µg ml^−1^). For each construct, separate expression trials were performed using three independently transformed colonies. A single colony was selected to inoculate 100 ml LB medium within 500 ml baffled shake flasks containing ampicillin and kanamycin. The culture was incubated at 310 K until the OD_600_ reached 0.5. Expression was then induced by the addition of 0.2% arabinose and 1 m*M* isopropyl β-d-1-thiogalactopyranoside (IPTG), and the culture was further incubated overnight at 310 K and 140 rev min^−1^. Finally, the culture was harvested by centrifugation.

### 2.3. Optimization of expression

To optimize the culture conditions for expression, the Box–Behnken experimental design (Box & Behnken, 1960 ▸) with three factors was conducted across 15 trials as follows: the induction OD_600_ (factor *A*: 0.5, 0.75 and 1), the arabinose concentration (factor *B*: 0.2%, 0.4% and 0.6%) and the induction temperature (factor *C*: 291, 301 and 310 K). To account for uncontrolled variability in the data, three centre-point experiments were included. The expression levels achieved under the optimal conditions were then compared across different growth media, including Terrific Broth (TB) and Superbroth (SB), and an alternative pCDFDuet expression system (Section 2.4). The study also explored how baffles, light conditions and the ratio of flask volume to culture volume affect the production of phytochromes.

### 2.4. Expression using the pCDFDuet vector

An alternative expression system for NPGP-H_6_, PGP-H_6_ and PG-H_6_ (Fig. 1 ▸) was also investigated by cloning them into pCDFDuet-1 (Novagen) using NcoI and HindIII. The resulting pCDFDuet-Sb.phyB ligation products (pCDFDuet–NPGP-H_6_, pCDFDuet–PGP-H_6_ and pCDFDuet–PG-H_6_) were co-transformed into competent *E. coli* BL21(DE3) cells (Novagen) along with p183 for PΦB or p171 for PCB co­production. Freshly transformed *E. coli* colonies were selected on LB plates with ampicillin (100 µg ml^−1^) and spectinomycin (50 µg ml^−1^). For large-scale production, colonies from the glycerol stock were used to inoculate 2 ml LB medium with suitable antibiotics in 10 ml culture tubes at 310 K and 140 rev min^−1^ to create starter cultures. The main cultures were grown by adding 400 µl of the starter culture to 400 ml SB medium with antibiotics as before in a 2 l baffled flask. Baffles are internal obstructions that help to increase the surface area of the fermenting substance, promoting better aeration and mixing. These cultures were grown at 310 K and 140 rev min^−1^ until an optimal OD_600_ was reached. The cultures were rapidly cooled in an ice–water bath for 30 min and holophytochrome production was then started by adding the optimized concentration of inducer. After induction, the cultures were grown for 16 h under the optimized temperature condition, harvested by centrifugation at 6000*g* and 277 K for 30 min, flash-frozen and stored at 193 K.

### 2.5. Expression with a TEV cleavage site

The pCDFDuet–PG-H_6_ construct was modified using the back-to-back primer PCR method to encode an N-terminal hexahistidine (His_6_) tag followed by a Tobacco etch virus (TEV) protease cleavage site (see Supplementary Table S1 for the primer sequences). This newly designed construct, pCDFDuet–H_6_-PG, serves as an additional strategy to alter the crystallization and diffraction properties of PG-H_6_.

### 2.6. Purification

The cell pellets were resuspended in lysis buffer [50 m*M* HEPES pH 7.8, 5 m*M* EDTA, 300 m*M* NaCl, 1 m*M* β-mercaptoethanol (β-ME)] and sonicated. The cell lysate was clarified by centrifugation and ice-cold ammonium sulfate buffer [50 m*M* Tris, 1 m*M* iminodiacetic acid (IDA), 3.3 *M* ammonium sulfate pH 7.8] was added to the supernatant at a volume ratio of three parts buffer to two parts supernatant. The mixture was stored on ice for 30 min and the precipitate was spun down at 277 K and 5000*g* for 30 min. The dark-green pellet was resuspended in binding buffer (50 m*M* HEPES pH 7.8, 500 m*M* NaCl, 1 m*M* IDA, 10 m*M* imidazole, 1 m*M* β-ME) and clarified at 277 K and 15 000*g* for 20 min. The supernatant was then applied onto a 10 ml Ni^2+^–NTA affinity column (Qiagen). The proteins were washed with 5 column volumes (CV) of binding buffer and then with 3 CV of 50 m*M* imidazole in binding buffer. The bound protein was eluted with 5 CV of 250 m*M* imidazole in binding buffer. Fractions displaying a green colour and a high *A*_660_/*A*_280_ specific absorbance ratio (SAR) were pooled for further analysis. Ni^2+^–NTA affinity purification was conducted under normal room lighting.

**Figure 2:**
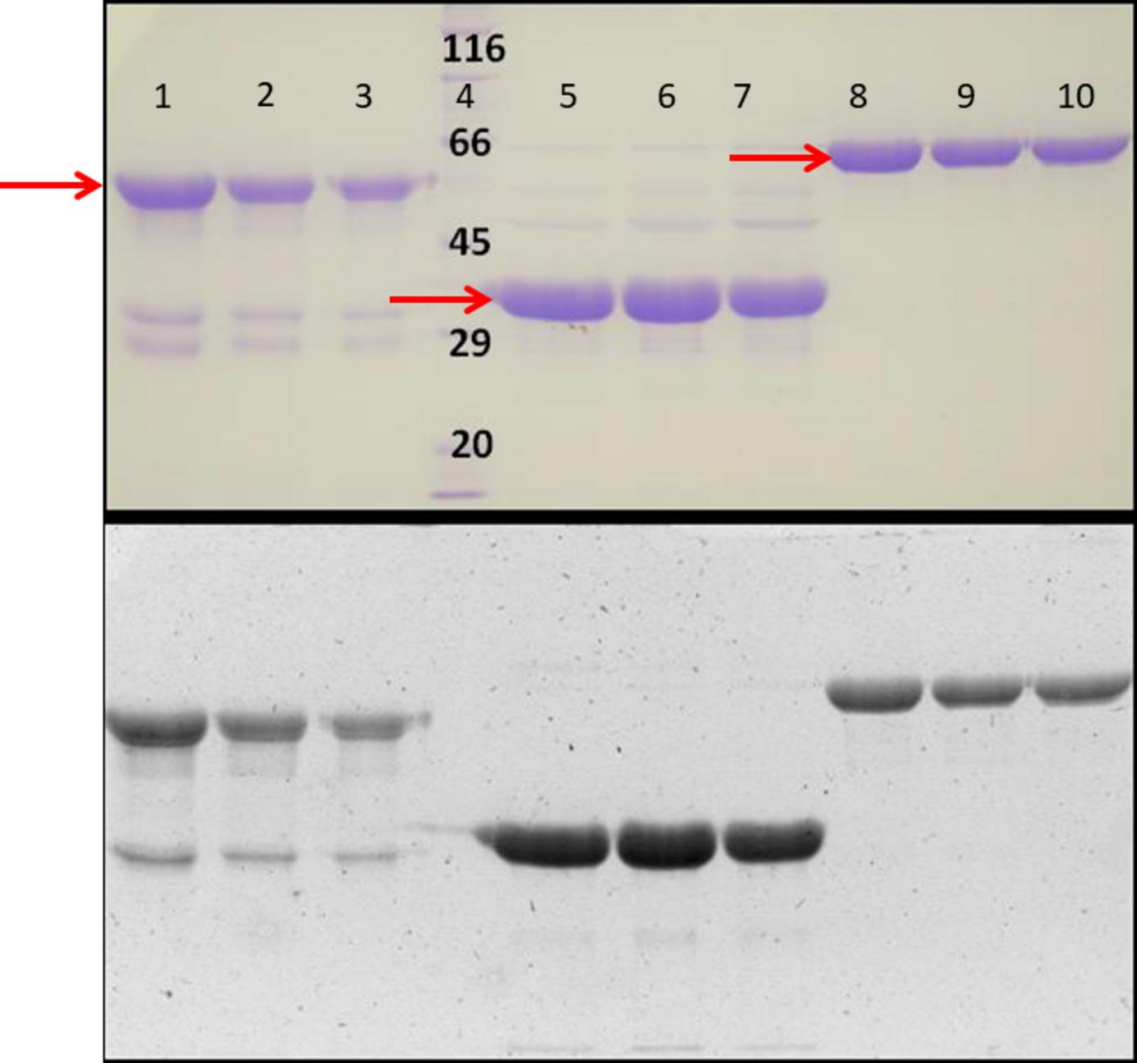
SDS–PAGE of the purified recombinant preparations of PGP-H_6_-PΦB, PG-H_6_-PΦB and NPGP-H_6_-PΦB in lanes 1–3, 5–7 and 8–10, respectively, detected by Coomassie staining (top) and Zn^2+^-induced fluorescence (bottom). Red arrows indicate the band associated with each purified protein. Molecular-mass markers are in lane 4, with sizes indicated in kDa.

Size-exclusion chromatography (SEC) experiments were conducted at room temperature using a Superdex 200 26/60 prep-grade column on an ÄKTAexplorer platform. Phytochrome samples were irradiated with FR before loading. The eluate was monitored at three wavelengths (280, 660 and 700 nm). Low-molecular-weight standards (GE Healthcare) were used for column calibration. Eluates were collected and analysed, and phytochrome fractions were pooled and stored at 277 K. The purified NPGP-H_6_-PΦB, PGP-H_6_-PΦB and PG-H_6_-PΦB preparations, as depicted in Fig. 2 ▸, were concentrated for spectroscopic characterization and crystallization trials. SEC was performed in a dark room using the safelight conditions produced by a 490 nm LED.

### 2.7. Purification of H_6_-PG and TEV cleavage

Affinity-purified H_6_-PG-PCB protein preparation was digested with TEV protease (Tropea *et al.*, 2009 ▸) at a ratio of 1 mg TEV protease per 200 mg phytochrome at 277 K to cleave the hexahistidine tag. The cleaved tag was subsequently removed by Ni^2+^–NTA affinity chromatography. The processed PG-PCB was subjected to SEC.

### 2.8. Crystallization

#### 2.8.1. Crystallization of NPGP-H_6_-PΦB

An initial crystallization screening was performed at 291 K using 96-well sitting-drop vapour-diffusion plates in conjunction with a Honeybee 963 robot (Genomic Solutions) and NeXtal suites (Qiagen). The screening procedure involved mixing 200 nl protein solution (20 mg ml^−1^ NPGP-H_6_-PΦB in 5 m*M* HEPES, 50 m*M* NaCl, 0.3 m*M* TCEP pH 7.8) with 200 nl crystallization reagent, followed by equilibration against 80 µl reservoir solution. The plates were irradiated with FR light and incubated in darkness. This process resulted in the lead condition 0.1 *M* CAPSO pH 9.5, 0.1 *M* LiSO_4_, 0.1 *M* NaCl, 12% PEG 4000. Additionally, small molecules from the Hampton Research Additive Screen HT kit were tested using the same approach. Manual optimization was carried out using 24-well plates (Sarstedt) in a hanging-drop setup, with each drop equilibrated against 500 µl reservoir solution at 291 K. In each crystallization trial, 1 µl 20 mg ml^−1^ protein solution prepared as above was combined with 1 µl reservoir solution, which contained the corresponding precipitant concentration and pH. The pH was varied from 9.25 to 10.5 in increments of 0.25 across six wells, while the PEG 4000 concentration was adjusted to four levels (7.5%, 10%, 12.5% and 15%) across four wells. This experiment was repeated using PEG 8000. In additional grid screening, the protein concentration was varied (20, 24, 28 and 32 mg ml^−1^) using 7.5% PEG 8000, maintaining the same pH range as before. To decrease the nucleation rate, the last experiment was replicated at 283 K. Crystals of the Pr state of NPGP-H_6_-PΦB were grown using the hanging-drop vapour-diffusion technique in 24-well plates (Sarstedt) at 291 K. A 1 µl mixture of the protein solution (24 mg ml^−1^ NPGP-H_6_-PΦB in 5 m*M* HEPES, 50 m*M* NaCl, 0.3 m*M* TCEP pH 7.8) was combined with 1 µl reservoir solution and equilibrated against 500 µl of the same reservoir solution (0.1 *M* CAPSO pH 9.3, 0.2 *M* NaCl, 1.5% glycerol, 0.03 *M* glycyl-glycyl-glycine, 7.5% PEG 8000). Crystallization, harvesting and cryoprotection procedures were carried out in a dark room using the safelight conditions produced by a 490 nm LED.

#### 2.8.2. Crystallization of PGP-H_6_-PΦB

Initial robotic crystallization screening using the same procedure as for NPGP-H_6_-PΦB at 291 K (Section 2.8.1) yielded several promising crystallization conditions, including (i) 0.1 *M* Tris–HCl pH 8.5, 0.2 *M* MgCl_2_, 30% PEG 4000, (ii) 0.1 *M* HEPES pH 7.5, 0.2 *M* MgCl_2_, 30% PEG 4000 and (iii) 0.1 *M* HEPES pH 7.5, 1.26 *M* ammonium sulfate. Additional screening with small molecules from the Hampton Research Additive Screen HT kit was also conducted. During manual optimization using 24-well plates (Sarstedt) in a hanging-drop setup, crystals were formed by PGP-H_6_-PΦB under the lead condition identified for NPGP-H_6_-PΦB. This condition was chosen as it is applicable to both constructs. The optimization process involved adjusting the protein concentration, pH and precipitant concentrations, similar to the approach used for NPGP-H_6_-PΦB. Crystals of the Pr state of PGP-H_6_-PΦB were grown using the hanging-drop vapour-diffusion technique in 24-well plates (Sarstedt). A 1 µl drop of protein solution (24 mg ml^−1^ PGP-H_6_-PΦB in 5 m*M* HEPES, 50 m*M* NaCl, 0.3 m*M* TCEP pH 7.8) was mixed with a 1 µl drop of reservoir solution (1 *M* CAPSO pH 9.3, 0.2 *M* NaCl, 1.5% glycerol, 0.03 *M* glycyl-glycyl-glycine, 7.5% PEG 8000). The mother liquor was allowed to equilibrate against 500 µl of the same reservoir solution at 291 K. All crystallization experiments were conducted under dim blue-green LED safelight. The plates were then wrapped in aluminium foil and incubated in darkness.

#### 2.8.3. Crystallization of PG-H_6_-PΦB and H_6_-PG-PCB

**Figure 3:**
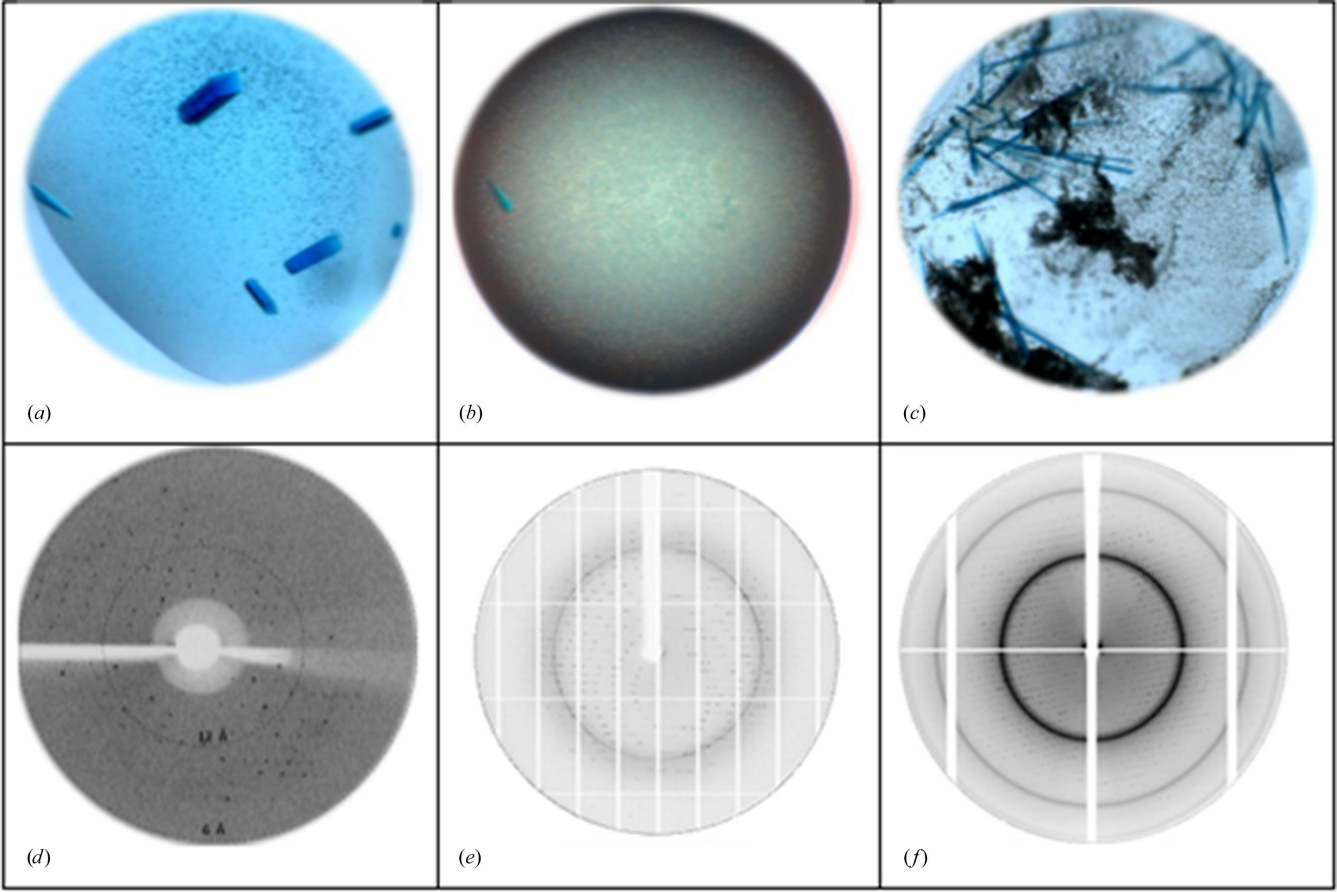
Blue crystals of the Pr forms of (*a*) NPGP-H_6_-PΦB, (*b*) PGP-H_6_-PΦB and (*c*) PG-H_6_-PΦB along with representative 1° oscillation diffraction images from the Pr crystals of (*d*) PGP-H_6_-PΦB, (*e*) PG-H_6_-PΦB and (*f*) H_6_-PG-PCB. The resolutions at the edges of these images correspond to 6, 2.4 and 1.8 Å, respectively.

Initial robotic crystallization trials were carried out using 96-well sitting-drop vapour-diffusion plates with a Honeybee 963 robot (Genomic Solutions) and NeXtal suites (Qiagen) at 291 K. Each trial involved mixing 200 nl of 10 mg ml^−1^ PG-H_6_-PΦB solution (in 5 m*M* HEPES, 50 m*M* NaCl, 0.3 m*M* TCEP pH 7.8) with 200 nl crystallization reagent, followed by equilibration against 80 µl reservoir solution. FR-illuminated plates were incubated in darkness. Needle-shaped PG-H_6_-PΦB crystals were obtained under the condition 0.05 *M* Tris–HCl pH 8.5, 0.5 *M* NaCl, 10% PEG 4000. The Hampton Research Additive Screen HT kit was also employed using a similar approach. Next, manual optimization of PG-H_6_-PΦB crystals was carried out using 24-well plates (Sarstedt) in a hanging-drop setup at 291 K. Protein concentrations (10, 15, 20 and 25 mg ml^−1^) and precipitant concentrations (7%, 8%, 9%, 10%, 11% and 12% PEG 4000) were systematically varied. For each condition, 1 µl PG-H_6_-PΦB solution, prepared as described earlier, was mixed with 1 µl of the corresponding reservoir solution. The resulting mixture was equilibrated against 500 µl of the same reservoir solution at 291 K. Needle-shaped dark green crystals appeared after two weeks using protein at 20 mg ml^−1^ in a solution consisting of 0.05 *M* Tris–HCl pH 8.5, 0.5 *M* NaCl, 0.5%(*w*/*v*) *n*-octyl-β-d-glucopyranoside, 9% PEG 4000. However, harvesting single crystals proved to be challenging due to the formation of tightly packed clusters (Fig. 3 ▸ *c*).

Hence, the protein concentration versus precipitant concentration grid-screening experiment was repeated at 283 K to reduce the nucleation rate and promote crystal growth. Further refinement of the buffer composition for protein preparation, the concentration of additive and the drop volume effectively addressed this issue, resulting in the production of usable crystals. To grow crystals of the Pr states of PG-H_6_-PΦB and H_6_-PG-PCB using the hanging-drop vapour-diffusion method in 24-well plates (Sarstedt), a 2 µl mixture of protein solution (20 mg ml^−1^ in 20 m*M* HEPES, 50 m*M* NaCl, 1 m*M* EDTA, 1 m*M* β-mercaptoethanol pH 7.8) was combined with 2 µl reservoir solution [0.1 *M* Tris–HCl pH 8.5, 0.5 *M* NaCl, 0.25%(*w*/*v*) *n*-octyl-β-d-glucopyranoside, 9% PEG 4000]. This mixture was then equilibrated against 500 µl of the same reservoir solution at 283 K.

### 2.9. Diffraction experiments

Various concentrations of cryoprotecting agents, including glycerol, ethylene glycol, 2-methyl-2,4-pentanediol (MPD), sucrose and low-molecular-weight PEGs, were introduced into the mother liquor to create cryosolutions for diffraction data-set collection under cryogenic conditions. Optimal cryo­protection for NPGP-H_6_-PΦB and PGP-H_6_-PΦB crystals was achieved by directly transferring them from the crystallization drop to 50%(*v*/*v*) crystallization solution supplemented with either 20%(*w*/*v*) glycerol or 25%(*w*/*v*) PEG 400. In contrast, supplementing 70%(*v*/*v*) reservoir solution with 70%(*w*/*v*) sucrose solution proved to be effective for cryoprotecting PG-H_6_-PΦB and H_6_-PG-PCB crystals. The crystals were allowed to equilibrate for 1 min, mounted in a nylon loop and then flash-cooled at 78 K in liquid nitrogen. X-ray diffraction measurements of NPGP-H_6_-PΦB, PGP-H_6_-PΦB and PG-H_6_-PΦB crystals were carried out on beamline 14.1 at the BESSY II synchrotron, Berlin, Germany using 0.898 Å wavelength X-rays. During data collection, diffraction images were acquired with 1° rotation steps and an exposure time of 1 s per image within a nitrogen gas stream at 100 K using an MX225 CCD detector. Data sets were processed with *XDS* using the *XDSAPP*2 graphical user interface (Sparta *et al.*, 2016 ▸). X-ray diffraction data sets for H_6_-PG-PCB were collected on beamline BM30A at the ESRF in Grenoble, France using a wavelength of 0.9677 Å.

## 3. Results

The *Sorghum* phyB DNA sequence was optimized for expression in *E. coli*, achieving a codon-adaptation index (CAI) of 0.85. The synthetic gene has 55.6% GC content and was modified to avoid mRNA secondary structures and specific restriction sites. The folding free energies of the first 12 codons of NPGP-H_6_ and PGP-H_6_ were calculated to be −19 and −36 kJ mol^−1^, respectively. In the pPROLar system, the myc tag and the ribosome-binding sequence (RBS) were eliminated through restriction digestion, whereas the RBS was reintroduced and a C-terminal 6×His tag was added by PCR.

PGP-H_6_-PΦB was successfully overexpressed using the pPROLar.A expression system at 303 K in LB medium from *E. coli* BL21_PRO_ cells at an OD_600_ of 0.5 with 0.2% arabinose and 1 m*M* IPTG overnight. *E. coli* BL21_PRO_ cells constitutively expresse *lac* and Tet repressors. The Box–Behnken design was used to optimize the production of PGP-H_6_-PΦB. A total of 15 expression trials were conducted, and the levels of PGP-H_6_-PΦB were quantified through difference spectra analysis. The optimal condition (BB4 in Supplementary Fig. S1) for PGP-H_6_-PΦB with the highest photoreversibility was found to be at 301 K with an induction OD_600_ of 1 and an arabinose concentration of 0.6%. However, no NPGP-H_6_-PΦB was detected using the pPROLar.A expression system.

To test an alternative expression system, the Sb.phyB constructs were cloned into the pCDFDuet vector at the MCS-I site using NcoI and HindIII restriction enzymes and the pPROLar constructs. Optimized protocols were developed for the production of NPGP-H_6_-PΦB, PGP-H_6_-PΦB and PG-H_6_-PΦB from pCDFDuet constructs with p183 in *E. coli* BL21(DE3) cells in SB. Optimum temperatures were 297 K for NPGP-H_6_-PΦB, 301 K for PGP-H_6_-PΦB and 303 K for PG-H_6_-PΦB. Optimal NPGP-H_6_-PΦB production used SB with glucose and glycerol at 297 K, an OD_600_ of 0.8 and 1 m*M* IPTG. The same induction OD and inducer concentration were used for maximum PGP-H_6_-PΦB and PG-H_6_-PΦB production. Adequate aeration was achieved with 400 ml culture volume in 2 l baffled flasks. Hence, NPGP-H_6_-PΦB, PGP-H_6_-PΦB and PG-H_6_-PΦB (Supplementary Table S2) were all successfully produced using the pCDFDuet expression system (see Table 1 ▸).

A three-phase purification strategy involving ammonium sulfate precipitation, Ni^2+^–NTA affinity chromatography and size-exclusion chromatography (SEC) was designed. SEC was used to ensure size homogeneity. No phytochrome was present in the void volume, indicating negligible aggregation for all preparations. The phytochromes eluted as single sharp peaks, suggesting monodispersion. All samples were slightly shifted from the expected positions for monomeric globular proteins based on their theoretical molecular weights, perhaps due to their elongated shapes or interactions with the column matrix. No degradation or aggregation was apparent even after storage at 277 K for ∼4 weeks. Purity was assessed by the *A*_660_/*A*_280_ SAR and the visualization of Zn^2+^-induced fluorescence and Coomassie staining after SDS–PAGE, as shown in Fig. 2 ▸. The SAR values for the optimized PGP-H_6_-PΦB and NPGP-H_6_-PΦB preparations after SEC were 1.2 and 1.4, respectively. This purity level was deemed to be sufficient for crystallization. The purified preparations were also used to study the effects of the NTE on the chromophore structure using resonance Raman spectroscopy (Velázquez Escobar *et al.*, 2017 ▸).

Crystals of NPGP-H_6_-PΦB and PG-H_6_-PΦB formed within two weeks and reached their maximum sizes in four weeks (Figs. 3 ▸ *a* and 3 ▸ *c*). PGP-H_6_-PΦB crystals formed immediately after the crystallization plates were set up and reached their maximum size overnight (Fig. 3 ▸ *b*). The NPGP-H_6_-PΦB crystals did not diffract X-rays. The crystals of PGP-H_6_-PΦB diffracted to 6–15 Å resolution with pronounced anisotropy (see Fig. 3 ▸ *d* and Table 2 ▸). In the absence of additives, the PG-H_6_-PΦB crystals diffracted to 3.5 Å resolution. Additive screening improved the resolution to 2.1 Å (Fig. 3 ▸ *e*). By moving the His tag to the N-terminus and substituting PΦB with PCB, we were able to produce H_6_-PG-PCB crystals that diffracted to a resolution of 1.8 Å (Fig. 3 ▸ *f*), providing detailed structural insights (Nagano *et al.*, 2020 ▸). Table 2 ▸ offers a concise overview of the main outcomes derived from the diffraction experiments, while Table 3 ▸ provides in-depth information on the crystallization conditions.

## 4. Discussion

Our study demonstrated notable differences in NPGP-H_6_-PΦB production when two different plasmids and host strains were employed. The pPROLar.A vector, which uses a hybrid *lac*/*ara* promoter and endogenous *E. coli* RNA polymerase, did not result in any detectable NPGP-H_6_-PΦB production. In contrast, substantial production was observed with the pCDFDuet-1 vector, which uses a T7 promoter for gene expression and is hosted in the *E. coli* BL21(DE3) strain, which produces T7 RNA polymerase. The differences in NPGP-H_6_-PΦB production between these two systems could be due to several factors. Firstly, the presence of the Tet repressor in *E. coli* BL21_PRO_ could suppress gene expression. Secondly, the T7 promoter is significantly stronger than the hybrid *lac*/*ara* promoter. Thirdly, T7 RNA polymerase is more efficient than the endogenous *E. coli* RNA polymerase. Lastly, the pCDFDuet-1 plasmid has a different ribosome-binding site (RBS) from pPROLar.A, which might improve translation of the NPGP-H_6_ mRNA. Other factors such as mRNA secondary structure could also contribute to the observed differences in NPGP-H_6_-PΦB production between the two plasmids. These results highlight the importance of experimenting with various plasmids and host strains for recombinant protein production, as the choice of expression system can significantly influence the protein yield.

Despite optimizing the induction OD_600_, inducer concentration and induction temperature, the production of NPGP-H_6_-PΦB from the pCDFDuet-1 vector was inconsistent, indicating the presence of additional influencing factors. One possible factor was oxygen availability. The enzyme heme oxygenase, which breaks down heme into biliverdin for chromophore synthesis, requires molecular oxygen. The impact of the geometry of baffled flasks on the production of recombinant phytochrome is frequently underestimated. In standard shake flasks, the transfer of molecular oxygen is dependent on the exposed surface area. In this study, we found that improving the aeration by increasing the surface area-to-volume ratio in baffled flasks was a key determinant in achieving consistent NPGP-H_6_ production. This consideration allowed a more reliable and reproducible production of NPGP-H_6_-PΦB.

The N-terminal extension (NTE) of Sb.phyB is predicted to be intrinsically disordered (Xue *et al.*, 2010 ▸). The cryo-electron microscopic structure of *Arabidopsis* phyB in the Pr state revealed a topologically complex dimeric organization (Li *et al.*, 2022 ▸). However, electron density was missing for both the NTE and the PAS1 domain. Both the PAS2 domain and the modulator loop in each protomer are crucial in maintaining the structural integrity and stability of the PHY domain. The PAS2 domain forms substantial contacts with the nPAS and GAF domains of the other protomer in the dimer, while the modulator loop wraps around the helical core of the PHY domain of its own protomer. Consequently, deleting the PAS repeat and downstream regions might release the PHY domain from structural restrictions, allowing greater mobility and thus fluctuations within the crystal lattice, which would in turn have a detrimental impact on the diffraction quality.

In *Sorghum* phyB, removing the flexible NTE and the PHY domain and shifting the hexahistidine tag, along with refinement of the crystallization buffer and cryoprotectant solutions, significantly improved both the crystallization propensity and the diffraction quality. Our results demonstrate the significance of taking into account the flexibility and mobility of protein domains when pursuing the crystallization of complex, multi-domain eukaryotic proteins. The practice of eliminating flexible and dynamic domains from a protein can aid in achieving high-resolution crystal structures. However, this method should be applied judiciously as it could potentially modify the structure and functionality of the remaining protein segments.

In *Sorghum* phyB, replacing PΦB with PCB improved the diffraction quality. The group attached to the C18 position of PΦB is a vinyl group, whereas in PCB it is an ethyl group. These groups differ in their conformational flexibility, spatial occupancy and electronic characteristics. Substituting PCB for the native PΦB chromophore in H_6_-PG-PCB crystals improved the diffraction quality, potentially by stabilizing the protein structure or altering the packing of protein molecules in the crystal. The ethyl group in PCB may help to immobilize the H_6_-PG protein and reduce the flexibility. Furthermore, considering the light-sensitive isomerization of the bilin chromophore during protein preparation, it is crucial to maintain a uniform and replicable safelight setting in darkness to prevent conformational heterogeneity and disruptions in crystal growth during the crystallization of phytochromes. The light-induced structural changes associated with photoconversion can introduce unintended crystal disorder and defects, significantly impacting the X-ray diffraction quality.

## Supplementary Material

Supplementary Tables and Figure. DOI: 10.1107/S2053230X24000827/nj5324sup1.pdf

## Figures and Tables

**Table 1:** Molecular-production information

Source organism	*MA3* from *Sorghum bicolor*, *HO1* and *pcyA* from *Synechocystis* 6803 and *HY2* from *Arabidopsis thaliana*
DNA source	*MA3* is a synthetic gene. *HO1*, *pcyA* and *HY2* are native DNA sequences.
Forward primer	See Supplementary Table S2
Reverse primer	See Supplementary Table S2
Cloning vector	pPROLar.A, pCDFDuet-1
Expression vector	pPROLar.A, pCDFDuet-1
Expression host	*E. coli* BL21_PRO_ (Clontech), *E. coli* BL21(DE3) (Novagen)
Complete amino-acid sequence of the construct produced	See Supplementary Table S1

**Table 2:** Summary of diffraction results

Construct	PGP-H_6_-PΦB	PG-H_6_-PΦB	H_6_-PG-PCB
Space group	*P*4_1_22	*P*3_1_21	*P*3_1_21
Resolution (Å)	6.0	2.1	1.8
*a*, *b*, *c* (Å)	122.54, 122.54, 310.19	133.88, 133.88, 46.95	134.74, 134.74, 46.54
α, β, γ (°)	90, 90, 90	90, 90, 120	90, 90, 120
PDB code	—	6tc5	6tby

**Table 3:** Summary of crystallization conditions

Constructs	NPGP-H_6_-PΦB and PGP-H_6_-PΦB	H_6_-PG-PCB and PG-H_6_-PΦB
Method	Hanging-drop vapour diffusion	Hanging-drop vapour diffusion
Plate type	Sarstedt 24-well plates	Sarstedt 24-well plates
Temperature (K)	291	283
Protein concentration (mg ml^−1^)	24	20
Buffer composition of protein solution	5 m*M* HEPES, 50 m*M* NaCl, 0.3 m*M* TCEP pH 7.8	20 m*M* HEPES, 50 m*M* NaCl, 1 m*M* EDTA, 1 m*M* β-ME pH 7.8
Composition of reservoir solution	0.1 *M* CAPSO pH 9.3, 0.2 *M* NaCl, 1.5% glycerol, 0.03 *M* glycyl-glycyl-glycine, 7.5% PEG 8000	0.1 *M* Tris–HCl pH 8.5, 0.5 *M* NaCl, 0.25%(*w*/*v*) *n*-octyl-β-D-glucopyranoside, 9% PEG 4000
Volume and drop ratio	2 µl (1 µl protein solution + 1 µl reservoir solution)	4 µl (2 µl protein solution + 2 µl reservoir solution)
Volume of reservoir (µl)	500	500
Light conditions	FR treatment followed by dark conditions and dim 490 nm blue-green LED safelight	FR treatment followed by dark conditions and dim 490 nm blue-green LED safelight
