# Supplementary material for: Expression, purification and crystallization of the photosensory module of phytochrome B (phyB) from Sorghum bicolor
Source: Acta Crystallogr F Struct Biol Commun. 2024 Feb 20;80(Pt 3):59–66. doi: 10.1107/S2053230X24000827 (PMC10910535; doi:10.1107/S2053230X24000827)
Supplement: Supplementary file 1 [file f-80-00059-sup1.pdf]

STRUCTURAL BIOLOGY COMMUNICATIONS

Volume 80 (2024)

Supporting information for article:

Expression, purification and crystallization of the photosensory module of phytochrome B (phyB) from *Sorghum bicolor*

Sintayehu Manaye Shenkutie, Soshichiro Nagano and Jon Hughes

Table S1 List of protein sequences used in the present study

Construct	Protein sequence
NPGP-H_6	MASGSRATPTRSPSSARPEAPRHAHHHHHHHSQSSGGSTSRAGGGGGGGGGGGGTAATATATATESVSKAVAQYTLDARLHAVFEQSGASGRSFDYSQSLRAPPTPSSEQQIAAYLSRIQRGGHIQPFGCTLAVADDSSFRLLAFSENAADLLDLSPHHSVPSLDSAAPPPVSLGADARLLFSPSSAVLLERAFAAREISLLNPLWIHSRVSSKPFYAILHRIDVGVVIDLEPARTEDPALSIAGAVQSQKLAVRAISRLQALPGGDIKLLCDTVVEHVRELTGYDRVMVYRFHEDEHGEVVAESRRDNLEPYLGLHYPATDIPQASRFLFRQNRVRMIADCHATPVRVIQDPGMSQPLCLVGSTLRAPHGCHAQYMANMGSIASLVMAVIISSGGDDEQTGRGGISSAMKLWGLVVCHHTSPRCIPFPLRYACEFLMQAFGLQLNMELQLAHQLSEKHILRTQTLLCDMLLRDSPTGIVTQSPSIMDLVKCDGAALYYHGKYYPLGVTPTESQIKDIIEWLTVCHGDSTGLSTDSLADAGYLGAAALGDAVCGMAVAYITPSDYLFWFRSHTAKEIKWGGAKHHPEDKDDGQRMHPRSSFKAFLEVVKSRSLPWENAEMDAIHSLQLILRDSFRDAAEGTSNSKAIVNGQVQLGHHHHHH
PGP-H_6	MAAYLSRIQRGGHIQPFGCTLAVADDSSFRLLAFSENAADLLDLSPHHSVPSLDSAAPPPVSLGADARLLFSPSSAVLLERAFAAREISLLNPLWIHSRVSSKPFYAILHRIDVGVVIDLEPARTEDPALSIAGAVQSQKLAVRAISRLQALPGGDIKLLCDTVVEHVRELTGYDRVMVYRFHEDEHGEVVAESRRDNLEPYLGLHYPATDIPQASRFLFRQNRVRMIADCHATPVRVIQDPGMSQPLCLVGSTLRAPHGCHAQYMANMGSIASLVMAVIISSGGDDEQTGRGGISSAMKLWGLVVCHHTSPRCIPFPLRYACEFLMQAFGLQLNMELQLAHQLSEKHILRTQTLLCDMLLRDSPTGIVTQSPSIMDLVKCDGAALYYHGKYYPLGVTPTESQIKDIIEWLTVCHGDSTGLSTDSLADAGYLGAAALGDAVCGMAVAYITPSDYLFWFRSHTAKEIKWGGAKHHPEDKDDGQRMHPRSSFKAFLEVVKSRSLPWENAEMDAIHSLQLILRDSFRDAAEGTSNSKAIVNGQVQLGHHHHHH
PG-H_6	MAAYLSRIQRGGHIQPFGCTLAVADDSSFRLLAFSENAADLLDLSPHHSVPSLDSAAPPPVSLGADARLLFSPSSAVLLERAFAAREISLLNPLWIHSRVSSKPFYAILHRIDVGVVIDLEPARTEDPALSIAGAVQSQKLAVRAISRLQALPGGDIKLLCDTVVEHVRELTGYDRVMVYRFHEDEHGEVVAESRRDNLEPYLGLHYPATDIPQASRFLFRQNRVRMIADCHATPVRVIQDPGMSQPLCLVGSTLRAPHGCHAQYMANMGSIASLVMAVIISSGGDDEQTGRGGISSAMKLWGLVVCHHTSPRCIPFPLRYACEFLMQAFGLQLNMELQLAHQLSEKHHHHHHH
H_6-TEV-PG	MHHHHHHENLYFQGAAYLSRIQRGGHIQPFGCTLAVADDSSFRLLAFSENAADLLDLSPHHSVPSLDSAAPPPVSLGADARLLFSPSSAVLLERAFAAREISLLNPLWIHSRVSSKPFYAILHRIDVGVVIDLEPARTEDPALSIAGAVQSQKLAVRAISRLQALPGGDIKLLCDTVVEHVRELTGYDRVMVYRFHEDEHGEVVAESRRDNLEPYLGLHYPATDIPQASRFLFRQNRVRMIADCHATPVRVIQDPGMSQPLCLVGSTLRAPHGCHAQYMANMGSIASLVMAVIISSGGDDEQTGRGGISSAMKLWGLVVCHHTSPRCIPFPLRYACEFLMQAFGLQLNMELQL

Table S2 List of primers used in the present study.

Direction	Sequence (5'- 3')	Purpose
F1	CCCGGTACC**<u>GAATTC</u>**ATTAAAGAGGAGAAATTAACCATGGCTTCTGGTTCT	Addition of EcoRI site for NPGP-H_6
F2	CGG**<u>GAATTC</u>***ttaaagaggagaaa*TTAACC***ATG***GctGCTGCTTACCTGTCTCGTA	Addition of EcoRI site, ribosome binding site and start codon for PGP-H_6 and PG-H_6
R1	GTAGGG**<u>AAGCTT</u>*****TTA***gtgatgatgatgatgatgACCCAGCTGAACCTGACC	Addition of HindIII site, stop codon and the 3'6xHis tag for NPGP-H_6 and PGP-H_6
R2	GTAGGG**<u>AAGCTT</u>*****TTA***gtgatgatgatgatgatgAGCCAGCTGCAGTTCCAT	Addition of HindIII site, stop codon and the 3'6xHis tag for PG-H_6
F3	TAATTAACCTAGGCTGCTGCCACC	Removal of the 3' 6xHis tag
R3	CAGCTGCAGTTCCATGTTCAGC	
F4	GAAAATCTGTATTTTCAAGGCGCTGCTTACCTGTCTCGTATCCAG	Addition of the 5' 6xHis tag and TEV site for H_6-TEV-PG
R4	GTGATGGTGATGGTGATGCATGGTATATCTCCTTATTAAAGTTAAACAAAATTATTTC	

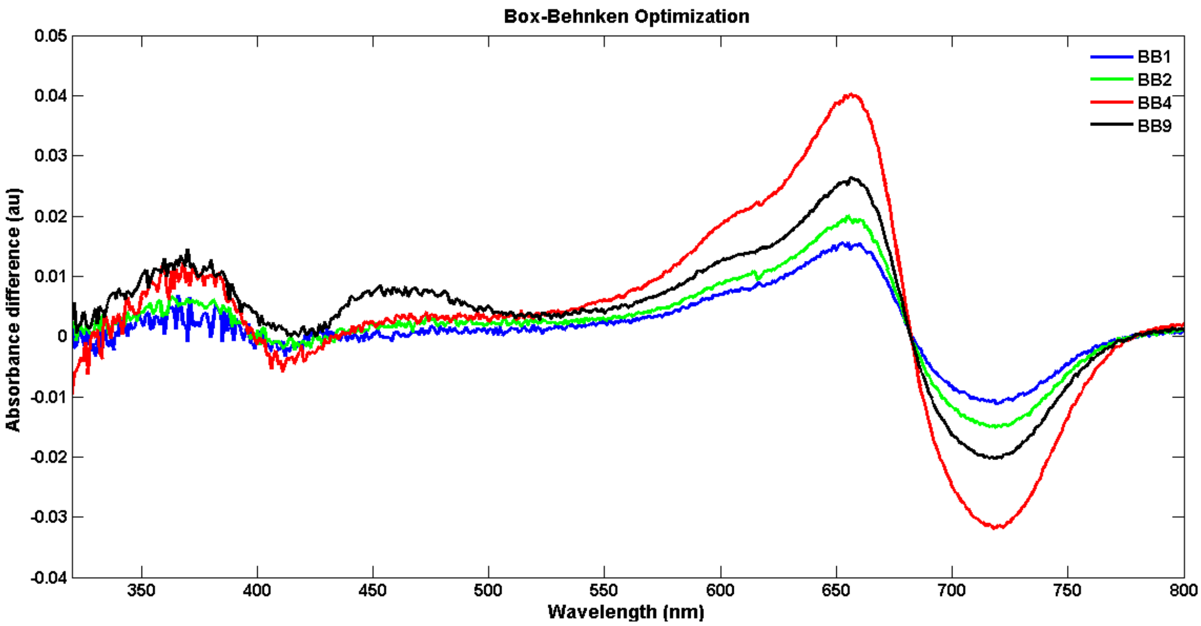

Figure S1 Representative difference spectra of the soluble fractions following the induction of PGP-H_6-PΦB holoprotein from pPROLar vector in conjunction with p183, across different temperatures, inducer concentrations, and induction OD values as part of the Box-Behnken optimization process.
